# DS0384 Alleviates Necrotizing Enterocolitis: Secretes N-carbamyl glutamic Acid and Participates in Lipid Metabolism and Lipid Peroxidation Processes

**DOI:** 10.4014/jmb.2410.10040

**Published:** 2025-02-13

**Authors:** Xiaofan Wei, Xiao Feng

**Affiliations:** Department of Paediatrics, Zhongshan Hospital of Xiamen University, No.201-209, Hubin South Road, Siming District, Xiamen 361009, P.R. China

**Keywords:** DS0384, fatty acid synthase, lipid peroxidation, necrotizing enterocolitis, toll-like receptor 4

## Abstract

Necrotizing enterocolitis (NEC) is a life-threatening inflammatory bowel disease linked to gut microbiome dysbiosis. This study evaluates the efficacy of *Limosilactobacillus reuteri* strain DS0384, which secretes N-carbamyl glutamic acid (NCG), in modulating lipid peroxidation and inflammatory pathways in NEC. After pretreatment with DS0384, NEC mouse model was induced by gavage with bacteria-containing formula. NCG levels in the ileum were measured via CE-TOFMS metabolomic analysis. Additionally, rat small intestinal epithelial IEC-6 cells were exposed to lipopolysaccharide (LPS), treated with DS0384 DNA (D-DNA), and/or transfected to overexpress fatty acid synthase (FASN) and Toll-like receptor 4 (TLR4). Lipid peroxidation, peroxidation and inflammatory factors and NF-κB pathways were analysed. Immunofluorescence was used to measure the expression levels of ZO-1 and TLR4 in the ileum. DS0384 treatment significantly reduced more histological abnormalities, apoptosis, and TLR4 expression in NEC mice, while restoring NCG levels, downregulating FASN and inhibiting lipid peroxidation and inflammation. Pre-treatment with D-DNA maintained cell vitality, reduced apoptosis, and suppressed TLR4/NF-κB-mediated inflammasome activation. Overexpression of FASN or TLR4 reversed these effects. DS0384 is a promising therapeutic against NEC, enhancing gut barrier integrity and modulating inflammatory and oxidative responses, suggesting potential clinical benefits in preventing NEC progression.

## Introduction

Necrotizing enterocolitis (NEC) is a life-threatening inflammatory gastrointestinal (GI) tract disease affecting preterm neonates, with an incidence that is inversely correlative to gestational age and high (>90%) in infants with a very low birth weight [1]. NEC accounts for an astounding rate of morbidity and mortality [2]. The most recognized etiological factors for NEC are prematurity and related events, which involve genetic predisposition, formula feeding, infections, premature immune system, intestinal immaturity, and microbial dysbiosis [2, 3]. Preterm neonates present several GI defense mechanisms underdeveloped, resulting in an increased susceptibility to GI injury and disease like NEC [3].

Oxidative stress occurs to contribute to NEC development due to immature and reduced antioxidant activity in preterm neonates [4]. It promotes inflammatory responses to induce cellular injury with lipid peroxidation in the bowel [5], leading to intestinal epithelial injury and barrier dysfunction [6]. In the injured immature intestinal barrier, a severe inflammatory response is triggered after bacterial translocation, and further enhances oxidant production, which incurs enterocyte apoptosis, necrosis and impaired enterocyte proliferation and epithelial repair to form NEC [4, 7, 8].

The intestinal epithelial barrier helps fend off the invasion by pathogenic bacteria [9]. Microbiota colonizing the gut interacts with the host to impact on the barrier development and maturation through facilitating intestinal epithelial cell proliferation and differentiation and maintaining epithelial junctions [10, 11]. Recently, transplantation of fecal microbiota containing a higher abundance of *Limosilactobacillus reuteri* into newborn preterm piglets has been discovered to decrease the risk of NEC [12]. DS0384, a novel strain of *L. reuteri*, has been shown to accelerate maturation of the fetal intestine, and this beneficial effect is uncovered to be attributed to its biologically active metabolite, N-carbamyl glutamic acid (NCG), which can reduce inflammatory phenotypes of human intestinal organoids and protects mice against dextran sodium sulfate-induced colitis [13]. These suggest that DS0384 may prevent NEC by secreting NCG to protect the intestinal epithelial barrier.

The Toll-like receptor 4 (TLR4) is an innate immune receptor that induces intense inflammatory response upon binding to endotoxin from bacteria [14]. Suppression of TLR4 inflammatory signaling pathway has been proposed as an effective therapy for NEC [2]. Through the Swiss database, we found that TLR4 ranked high among the targets of NCG. Accordingly, NCG produced from DS0384 is presumed to inhibit TLR4-mediated inflammatory responses in NEC.

In addition, inhibiting the TLR4-NF-κB pathway subdues both lipid peroxidation and inflammasome activation in hyperlipidemia [15]. NCG can decrease the transcription level of fatty acid synthesis-related genes, FASN, in visceral adipocytes of Japanese seabass [16]. Since lipid peroxidation is implicated in NEC development [17], we speculated that DS0384 antagonizes lipid peroxidation and inflammation responses through downregulating FASN and inhibiting the TLR4-NF-κB-mediated inflammasome activation, thereby alleviating NEC.

The present established both *in vivo* and *in vitro* NEC models to verify the correctness of the above reasoning.

## Methods and Materials

### Ethics Statement

All animal protocols were approved by the Ethics Committee of Zhejiang Baiyue Biotech Co., Ltd. for Experimental Animals Welfare (Approval number: ZJBYLA-IACUC-20221202), and the animal experiment was conducted in strict accordance with the guidelines of the National Institutes of Health on Animal Care and Use.

### DS0384 Gavage and NEC Induction in Neonatal Mice

Neonatal male C57BL/6 mice (*n* = 30), aged 7–10 days, weighing 5-8 g, were maintained under a controlled condition comprising 22 ± 1.0°C, 50 ± 5% humidity, and a 12/12 h light/dark circadian cycle, and given free access to standard rodent house chow diet and water. Experimental NEC was induced by feeding the mice via gavage five times per day for 4 days with Similac Advance infant formula (Abbott Nutrition, USA) supplemented with intestinal bacteria from stool slurry created by a NEC infant (1 ml formula contains 12.5 μl original stool slurry) [18]. Meanwhile, the mice were subjected to a 10-min hypoxia (5% O_2_-95% N_2_) insult using a hypoxic chamber twice a day for a total of 4 days [18]. DS0384, which belongs to the Bio R&D Product program (https://biorp.kribb.re.kr/), was obtained from the Korean Collection for Type Cultures (KCTC, Republic of Korea). Gavage with DS0384 (5 × 10^6^ CFU) was performed once daily, 4 days before NEC induction, while gavage with 200 μl phosphate buffered saline (PBS, Sigma-Aldrich, USA) served as the control treatment [13]. Right after NEC induction, all the mice were anesthetized using 1% pentobarbital sodium (P010, 50 mg/kg, Sigma-Aldrich), and euthanized via cervical dislocation. Mouse ileum was harvested for analysis.

### Grouping

Con, NEC and DS0384 groups were set up, with 10 mice randomly distributed in each group. In NEC group, mice received NEC induction, and the mice from the DS0384 group underwent DS0384 gavage and NEC induction. PBS gavage was performed for the mice in the control group.

### Hematoxylin-Eosin (H&E) Staining

Mouse ileum was fixed with 4% paraformaldehyde (P885233, MACKLIN, China) for 24 h, after which dehydration using gradient ethanol, hyalinization via xylene (95682, Sigma-Aldrich) and paraffinization (1496904, Sigma-Aldrich) was conducted. Paraffinized mouse ileum was sectioned onto 4-μm-thick slices. After being deparaffinized via xylene and rehydrated using gradient ethanol, the ileal slices were stained with hematoxylin (HY-N0116, MedChemExpress, USA) for 8 min. 1% hydrochloric alcohol was then used to differentiate the slices. Developing blue was performed using weakly alkaline water (67362, Sigma-Aldrich). Later, the slices were dyed with eosin (HY-D0505A, MedChemExpress) for 5 min. Dehydration and hyalinization were conducted with the slices followed by sealing with neutral balsam (N861409, Macklin, China). Histological ileal lesions were observed by an optical microscope (ZEISS Primotech, Carl Zeiss, Germany) under ×100 magnification, and the lesions were scored by a scoring system, with less histological abnormalities indicating a higher score (0 = best, 6 = worst).

### Immunohistochemical Aassay

After deparaffinization and rehydration, ileal slices were treated with 3% H_2_O_2_ to quench endogenous peroxidase activities. Then, antigens were retrieved by microwaving the slices along with 1 mol/l citrate buffer (C9999, Sigma-Aldrich) for 10 min. Later, the slices were blocked using Ultra V block (Lab Vision Corporation, USA) at 37°C for 5 min, followed by incubation with primary antibody for caspase3 (EPR18297, Abcam, UK) at 4°C overnight. Secondary antibody HRP-conjugated goat anti-rabbit IgG (31460, Thermo Fisher Sxientific, USA) was used to probe caspase3 via incubation for 1 h. DAB solution (D8001, Sigma-Aldrich) was added for an observable immune reaction. Nuclear counterstaining was performed with hematoxylin (H9627, Sigma-Aldrich). Finally, areas positive for caspase3 were observed by the optical microscope under ×100 magnification.

### Metabolome Analysis

Fresh mouse ileum was homogenized and centrifuged for 3,000 ×*g* for 10 min to obtain supernatant, which was mixed with 20 μl of Milli-Q water to extract ionic metabolites. Metabolome analysis of samples was carried out CE-TOFMS aided with the HMT Basic Scan package, as previously described [13]. An Agilent CE capillary electrophoresis system equipped with a time-of-light mass spectrometer (Agilent 6210, Agilent Technologies, USA) was utilized to perform CE-TOFMS analysis, with Agilent G2201AA ChemStation software (version B.03.01, Agilent Technologies) used for system control.

### Immunofluorescence Assay

Paraffinized ileal slices were rehydrated, followed by antigen retrieval and endogenous peroxidase elimination as indicated above. Subsequently, the slices were blocked with Ultra V block for 5 min and incubated with antibodies for TLR4 (ab22048, Abcam) and ZO-1 (ab221547, Abcam) at 4°C overnight. Later, the slices were subjected to incubation with Alexa Fluor™ 647-labeled anti-mouse/rabbit secondary antibodies (A-21235/A-31573, Thermo Fisher Scientific). 4’6-diamidino-2-phenylindole dihydrochloride (DAPI; 62247, Thermo Fisher Scientific) was used to stain nuclei. Fluorescent signals were detected by a fluorescence microscopy (Eclipse Ni, Nikon, Japan) under ×400 magnification.

### Cell Culture

Rat small intestine epithelial IEC-6 cell lines were purchased from American Type Culture Collection (ATCC)(CRL-1592, USA), and grown in Dulbecco's Modified Eagle's Medium (30-2002, ATCC, USA), supplemented with 0.1 U/ml human insulin (I2643, Sigma-Aldrich) and 10% fetal bovine serum (FBS; HY-P2352, MedChemExpress) with 5% CO_2_ at 37°C.

### Cell Transfection

FASN overexpression plasmids (RR^2^12398, OriGene, USA) and TLR4 overexpression plasmids (RR^2^12307, OriGene, USA) as well as negative control for the overexpression plasmids (NC; PS100001, OriGene) were used to transfect IEC-6 cells with the help of Lipofectamine 3000 transfection reagent (L3000015, Thermo Fisher Scientific). Simply put, after being inoculated in 96-well plates (1 × 10^4^ cells/well), IEC-6 cells were cultured to reach 80% confluency and incubated with gene-lipid complexes prepared by incubating the above plasmids (0.2 μg) and Lipofectamine 3000 transfection reagent (0.45 μl) with Opti-MEM Medium and P3000 Reagent for 10 min at 37°C. 48 h later, the transfected cells were transferred to quantitative reverse transcription-polymerase chain reaction (qRT-PCR) to assess the transfection efficiency.

### Bacterial DNA Isolation

Bacterial DNA (D-DNA) was isolated from the probiotic DS0384 using a modified approach based on the “Bust n’ Grab” technique [19] according to the previous described protocols [18]. Briefly, lyophilized material was washed by nuclease-free water, and then lysed using 10 mg/ml lysozyme (10837059001, Sigma-Aldrich), following which three rapid freeze-thaw cycles (from −60°C up to 90°C) was conducted. Equal volumes of phenol:chloroform:isoamyl alcohol (25:24:1) were used to isolate D-DNA, which was then extracted using an equal volume of chloroform:isoamyl alcohol (24:1), followed by precipitation in pre-cooled 100% ethanol and 3 M sodium acetate (2:0.3). After being treated with 100 μg/ml RNase A (RNASEA-RO, Sigma-Aldrich) and 200 μg/ml Proteinase K (1.07393, Sigma-Aldrich, USA), the concentration of D-DNA was determined at 260 nm and 260/ 280 nm ratio using a spectrophotometer (NanoDrop 2000, Thermo Fisher Scientific) as well as through agarose gel electrophoresis [18]. The spectrometer scans from m/z 50 to 1000, extracting peaks using MasterHands automatic integration software (Keio University, Japan) to obtain peak information, including m/z, peak area and migration time. The remaining peaks were labeled according to the HMT metabolite database, and the area of the labeled peaks was normalized into internal standard and sample volume to obtain the relative levels of each metabolite.

### Cell Treatment

IEC-6 cells, whether transfected or not, were treated with 75 μg/ml D-DNA, and 1 h later, the cells were exposed to 10 μg/ml LPS (L2630, Sigma-Aldrich) for 1 h to induce cellular injury models [18]. The purpose of pretreatment with D-DNA is to simulate the effect of DS0384 on cellular injury models.

### Cell Counting Kit (CCK)-8 Assay

IEC-6 cells, whether transfected or not, were pretreated with D-DNA, exposed to LPS, and then seeded in 96-well plates (2 × 10^3^ cell/well), and cultured to adhere to the well. CCK-8 reagent (10 μl, CA1210, Solarbio, China) was added per well. After a 2-h incubation at 37°C, the cells were subjected to absorbance measurement using the microplate reader at 450 nm.

### Flow Cytometry

Annexin V-FITC Early Apoptosis Detection kit (#6592, Cell Signaling Technology, USA) was utilized to detect the apoptosis of IEC-6 cells that underwent D-DNA pretreatment and LPS exposure in the presence of transfection or not. In brief, the processed cells (1 × 10^6^) were collected after centrifugation at 2,000 ×*g* for 10 min, and then washed with pre-cooled PBS. Later, the cells were resuspended in Annexin-V binding buffer to obtain cell suspension, 96 μl of which was incubated with 1 μl Annexin V-FITC conjugate and 12.5 μl propidium iodide solution for 10 min protected from light. After being suspended with Annexin-V binding buffer, the cells were transferred to a flow cytometer (Cytoflex, Beckman Coulter, USA).

### Determination of Lipid Peroxidation-Related Marker Levels

The levels of advanced oxidation protein products (AOPP) and lactoperoxidase (LPO) in the mice’s ileum and in the supernatant of IEC-6 cells that had undergone D-DNA pretreatment and LPS exposure in the presence of transfection or not were determined using colorimetry-based assay kits (ab242295 and ab133085, Abcam). In short, cell supernatant was obtained via centrifugation at 2,000 ×*g* for 20 min, and the supernatant of homogenized ileum was harvested as the procedure in metabolome analysis. For AOPP assay, the supernatant (200 μl) was added into antibody-coated 96-well plates, followed by incubation with 10 μl Chloramine Initiator Solution for 5 min at room temperature on a shaker. Later, 20 μl of Stop Solution was added, and immediately the cell absorbance was read by the microplate reader at 340 nm. For LPO assay, 500 μl of the chloroform extract (lipid hydroperoxides) of the supernatant was mixed with 450 μl of chloroform-methanol solvent mixture, and added with 50 μl of the freshly prepared Chromogen. After the assay was kept for 5 min, 300 μl of the reactant was added into each well of 96-well plates and subjected to the microplate reader for absorbance measurement at 500 nm. Oxidative stress index (OSI) was calculated according to the formula: OSI (arbitrary unit) = TOS (μmol H_2_O_2_ equivalent/g protein)/TAS (mmol Trolox equivalent/g protein), as previously described [5].

### qRT-PCR

Total RNA was extracted from mice’s ileum and processed IEC-6 cells with Trizol reagents (15596026, Thermo Fisher Scientific), followed by quantification using the spectrophotometer. cDNA was synthesized by reverse-transcribing RNA (5 μg) employing reverse transcription kits (K1622, Yaanda Biotechnology, China). A PCR detection System (LightCycler 96, Roche, USA) added with Eastep qPCR Master Mix (LS2062, Promega, USA) was used for quantitative PCR analysis. The reaction condition was set as: 95°C for 10 min, followed by 40 cycles of 95°C for 15 s and 60°C for 1 min, and the used primers were listed in Table S1. The mRNA expression values were calculated via the 2^-ΔΔCt^ method [20] and GAPDH served as the internal control.

### Western Blot

Total protein was isolated from mice’s ileum and processed IEC-6 cells with RIPA Lysis Buffer (20-188, Sigma-Aldrich), followed by the assessment of protein concentration assessed using a BCA kit (A53227, Thermo Fisher Scientific). The protein was separated on SDS-PAGE gel electrophoresis (1615100, Bio-Rad), and then transferred onto a polyvinylidene fluoride (PVDF) membrane (03010040001, Sigma-Aldrich), which was later blocked in 5%skimmed milk for 2 h at room temperature. The membrane was incubated overnight at 4°C with primary antibodies for TLR4 (#14358, 120 kDa, 1:1,000, Santa Cruz, USA; ab217274, 130 kDa, 1:1,000, Abcam, UK), NF-κB (ab16502, 60 kDa, 0.5 μg/ml, Abcam), NLR family pyrin domain containing 3 (NLRP3; ab263899, 118 kDa, 1:1,000, Abcam), cleaved (c)-caspase3 (#9664, 17 kDa, 1:1,000, Cell Signaling Technology, USA) and GAPDH (ab8245, 37 kDa, 1:500, Abcam). Subsequently, after being washed twice with T20 TBS (37571, Thermo Fisher Scientific), the membrane was added with anti-Rabbit/Mouse secondary antibodies (ab97051/ab6789, Abcam, UK) and incubated for 2 h at room temperature. Specific protein bands were visualized with Luminol Reagent (sc-2048, Santa Cruz) on a luminescence imager (LAS4000, Fujifilm, Japan), and the band density was analyzed using ImageJ software (3.0 version, National Institutes of Health, USA).

### Statistical Analysis

Results were obtained from experiments repeated at least thrice and expressed as mean ± standard deviation (SD). Statistical analysis was performed using Graphpad prism (version 8.0, GraphPad Software Inc., USA). Comparison of multiple experimental groups was implemented adopting one-way analysis of variance (ANOVA). Statistical significance was set at *p* < 0.05.

## Results

### DS0384 Gavage Ameliorated Histological Lesions and Apoptosis, Restored NCG Content, Downregulated FASN, and Inhibited Lipid Peroxidation and Inflammation In the Ileum of NEC Mice

**Fig. 1:**
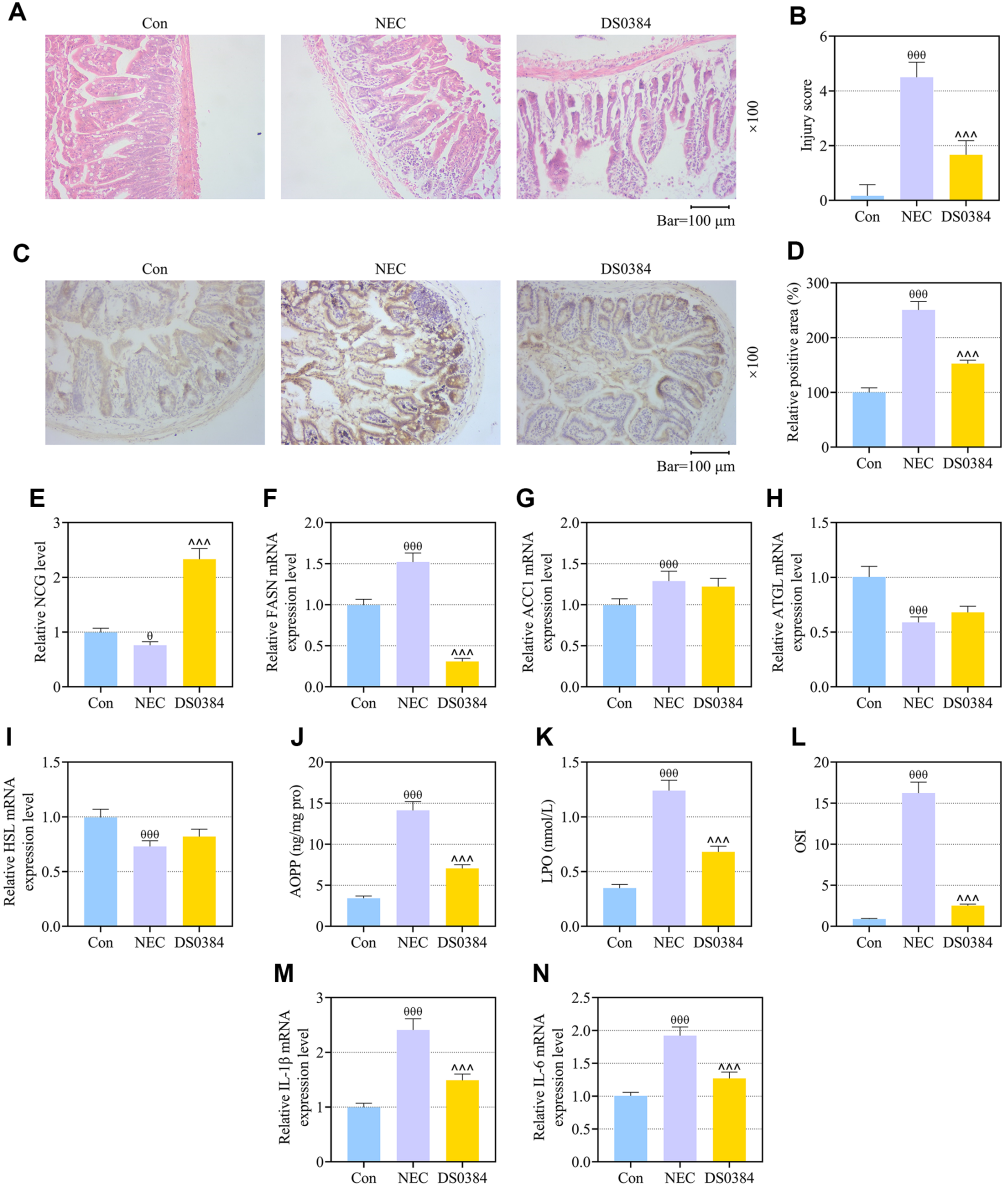
DS0384 gavage ameliorated histological lesions and apoptosis, restored NCG content, downregulated FASN, and inhibited lipid peroxidation and inflammation in the ileum of NEC mice. (**A-E, H-N**) Neonatal mice underwent NEC induction by being fed via gavage for 4 days with formula rich in intestinal bacteria in stool slurry created by a NEC infant (1 ml formula contains 12.5 μl original stool slurry), and 4 days before NEC induction, mice received gavage with DS0384 (5 × 10^6^ CFU). (**A, B**) Ileal histological lesions were observed via hematoxylin-eosin staining (magnification: ×100; scale bar: 100 μm), and the related injury scores were presented. (**C, D**) The areas positive for caspase3 in the ileum were determined by immunohistochemical assay (magnification: ×100; scale bar: 100 μm). (**E**) The content of NCG in the ileum was assessed via CE-TOFMSbased metabolic analysis. (**F-I**) The expressions of FASN, ACC1, ATGL and HSL in the ileum were analyzed using qRT-PCR, with GAPDH serving as the internal control. (**J-L**) The levels of AOPP and LPO in the ileum were measured via the colorimetric method and OSI was calculated. (**M, N**) The expressions of IL-1β and IL-6 in the ileum were analyzed using qRT-PCR, with GAPDH serving as the internal control. ^θ^ vs. Con, *p* < 0.05, ^θθθ^ *p* < 0.001; ^^^ vs. NEC, *p* < 0.001. (NEC, necrotizing enterocolitis; NCG, N-carbamyl glutamic acid; ACC1, acetyl- CoA carboxylase 1; ATGL, patatin like phospholipase domain containing 2; HSL, hormone-sensitive lipase; AOPP, advanced oxidation protein products; LPO, lactoperoxidase; OSI, oxidative stress index; IL-1β/IL-6, interleukin-1β/interleukin-6; qRT-PCR, quantitative reverse transcription polymerase chain reaction).

Feeding with formula rich in intestinal bacteria of a NEC infant was conducted to establish NEC mice, whose ileum was observed through H&E staining to show lesions with an extremely high injury score (Fig. 1A and 1B, *p* < 0.001). Meanwhile, immunohistochemical assay revealed that the area positive for caspase3 in the ileum of mice expanded following treatment with the NEC intestinal bacteria-enriched formula (Fig. 1C-1D, *p* < 0.001). Gavage with DS0384 resulted in decreases in the injury score and reduction of the caspase3 positive area in the ileum of NEC mice (Fig. 1A-1D, *p* < 0.001). Metabolomic profiles using CE-TOFMS showed a reduced content of NCG in the ileum of NEC mice compared to that content in the ileum of control mice (Fig. 1E, *p* < 0.05); yet the addition of DS0384 predominantly reversed that reduction of the NCG content (Fig. 1E, *p* < 0.001). In the NEC mice’s relative to control mice’s ileum, the expressions of FASN and ACC1 increased and those of ATGL and HSL diminished, as shown by qRT-PCR (Fig. 1F-1I, *p* < 0.001); notably, only the NEC-related increase in the expressions of FASN in mice’s ileum was reversed when DS0384 was given (Fig. 1F, *p* < 0.001). Colorimetric assay displayed that the levels of lipid peroxidation-related markers, AOPP, LPO and OSI ascended in the ileum of NEC mice, contrasted with those in control mice’s ileum (Fig. 1J-1L, *p* < 0.001). Compared with PBS treatment, DS0384 gavage decreased the level of these lipid peroxidation markers in the ileum of NEC mice (Fig. 1J-1L, *p* < 0.001). Moreover, NEC mice in contrast with control mice exhibited elevated expressions of IL-1β and IL-6 in their ileum (Fig. 1M-1N, *p* < 0.001); however, the elevation of the level of these inflammatory cytokines weakened with DS0384 gavage (Fig. 1M-1N, *p* < 0.001).

### DS0384 Gavage Regained ZO-1 Expression and Repressed TLR4 Expression in the Ileum of NEC Mice

**Fig. 2:**
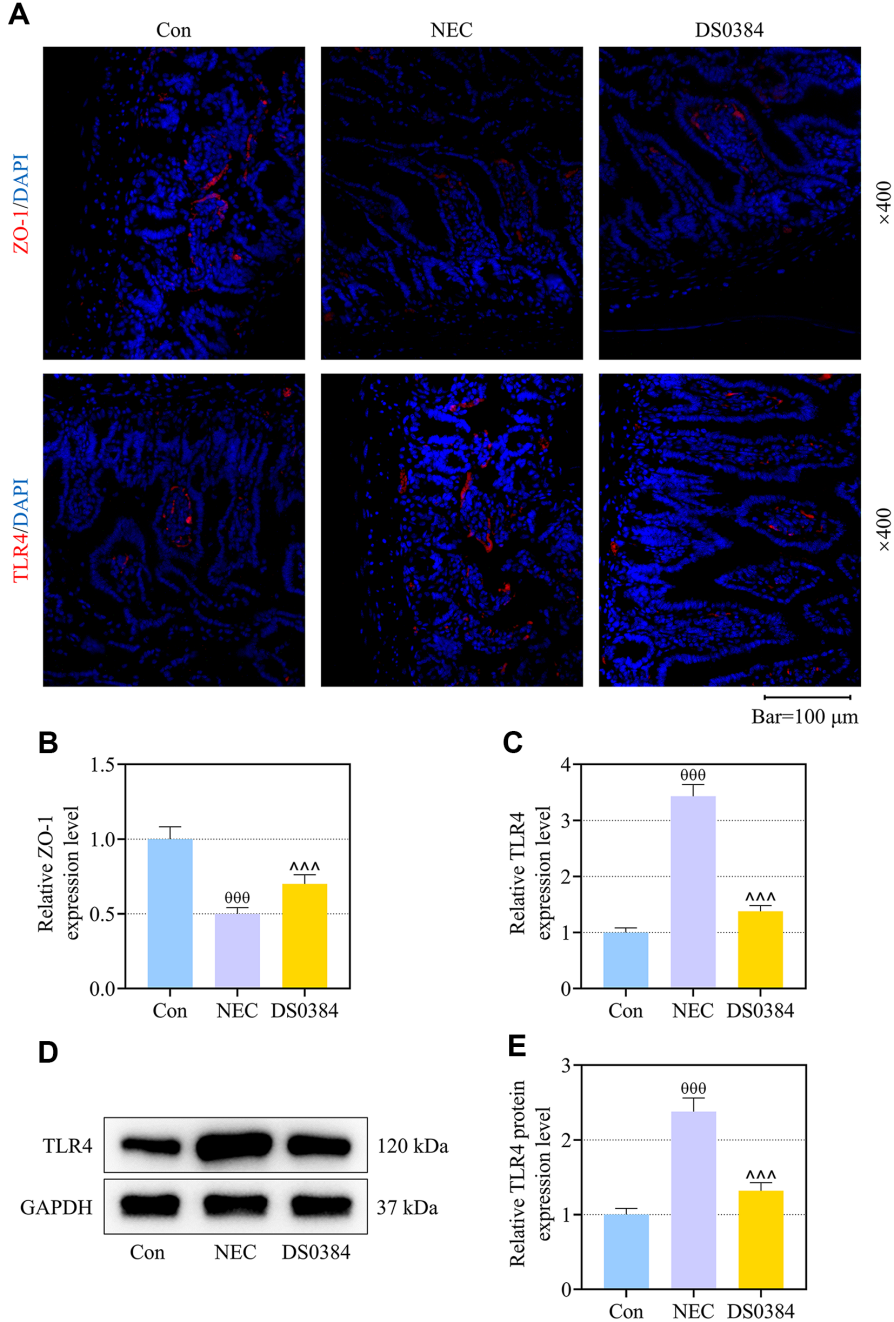
DS0384 gavage regained ZO-1 (tight-junction associated protein) expression and repressed TLR4 expression in the ileum of NEC mice. (**A-E**) Neonatal mice underwent NEC induction by being fed via gavage for 4 days with formula rich in intestinal bacteria in stool slurry created by a NEC infant (1 ml formula contains 12.5 μl original stool slurry), and 4 days before NEC induction, mice received gavage with DS0384 (5 × 10^6^ CFU). (**A-C**) The expressions of TLR4 and ZO-1 in the ileum were determined through immunofluorescence assay (magnification: ×400; scale bar: 100 μm). (**D, E**) The expression of TLR4 in the ileum was analyzed using western blot, with GAPDH serving as the internal control. ^θθθ^ vs. Con, *p* < 0.001; ^^^ vs. NEC, *p* < 0.001. (NEC, necrotizing enterocolitis; TLR4, Toll-like receptor 4; qRT-PCR, quantitative reverse transcription polymerase chain reaction).

ZO-1 is a tight-junction associated protein involved in the maintenance and regulation of epithelial palisade and barrier function [21]. Immunofluorescence assay illustrated that the expression of ZO-1 lessened while that of TLR4 increased in the ileum of mice after feeding with the NEC intestinal bacteria-enriched formula (Fig. 2A-2C, *p* < 0.001). Treatment with DS0384 augmented expression of ZO-1 and micrified that of TLR4 in the ileum of NEC mice (Fig. 2A-2C, *p* < 0.001). Western blot produced a similar result related to the TLR4 expression, showing that the NEC intestinal bacteria-enriched formula increased the protein expression of TLR4 in mice’s ileum, which yet was attenuated by DS0384 gavage (Fig. 2D-2E, *p* < 0.001).

### D-DNA Pretreatment Preserved Viability, Inhibited Apoptosis and Downregulated FASN in LPS-Exposed IEC-6 Cells

**Fig. 3:**
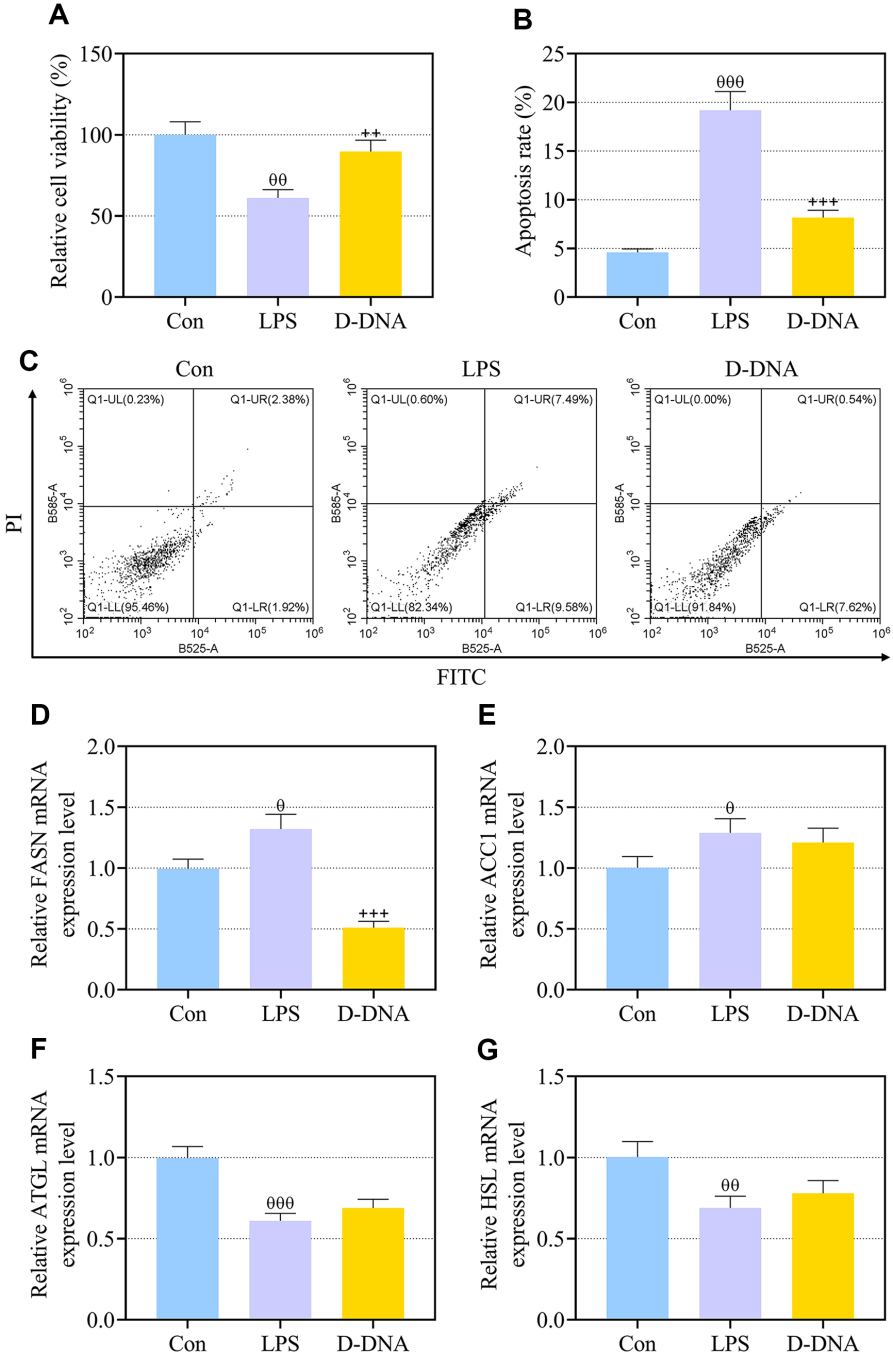
D-DNA pretreatment preserved viability, inhibited apoptosis and downregulated FASN in LPS-exposed IEC-6 cells. (**A-G**). IEC-6 cells were exposed to LPS for 1 h before a 1-h administration with 75 μg/ml DNA from DS0384 (D-DNA). (**A**) The viability of IEC-6 cells was measured by cell counting kit-8 assay. (**B, C**) The apoptosis of IEC-6 cells was detected through flow cytometry. (**D-G**) The expressions of FASN, ACC1, ATGL and HSL in IEC-6 cells were analyzed using qRT-PCR, with GAPDH serving as the internal control. ^θ^ vs. Con, *p* < 0.05, ^θθ^ *p* < 0.01, ^θθθ^*p* < 0.001; ^++^ vs. LPS, *p* < 0.01, ^+++^ *p* < 0.001. (NEC, necrotizing enterocolitis; LPS, lipopolysaccharide; PI, propidium iodide; FITC, fluorescein isothiocyanate; ACC1, acetyl-CoA carboxylase 1; ATGL, patatin like phospholipase domain containing 2; HSL, hormone-sensitive lipase; qRT-PCR, quantitative reverse transcription polymerase chain reaction).

DNA from DS0384 was isolated as D-DNA and used to pretreat IEC-6 cells, which were later induced into cell injury models via LPS stimulation. According to CCK-8 assay, LPS stimulation caused decreases in the viability of IEC-6 cells (Fig. 3A, *p* < 0.01), whereas D-DNA pretreatment restored the viability of LPS-induced IEC-6 cells (Fig. 3A, *p* < 0.01). As shown through flow cytometry, apoptosis of IEC-6 cells was elicited by LPS (Fig. 3B and 3C, *p* < 0.001), which was partly abrogated with the addition of D-DNA (Fig. 3B and 3C, *p* < 0.001). QRT-PCR denoted that FASN and ACC1 expressions arose and ATGL and HSL expressions declined in IEC-6 cells due to LPS stimulation (Fig. 3D-3G, *p* < 0.05). The reversal effect of D-DNA on was observed merely on LPS-induced increase in the expression of FASN in IEC-6 cells (Fig. 3D, *p* < 0.001).

### D-DNA Pretreatment Inhibited Lipid Peroxidation, Inflammation and TLR4/NF-κB Signaling in LPS-Exposed IEC-6 Cells

**Fig. 4:**
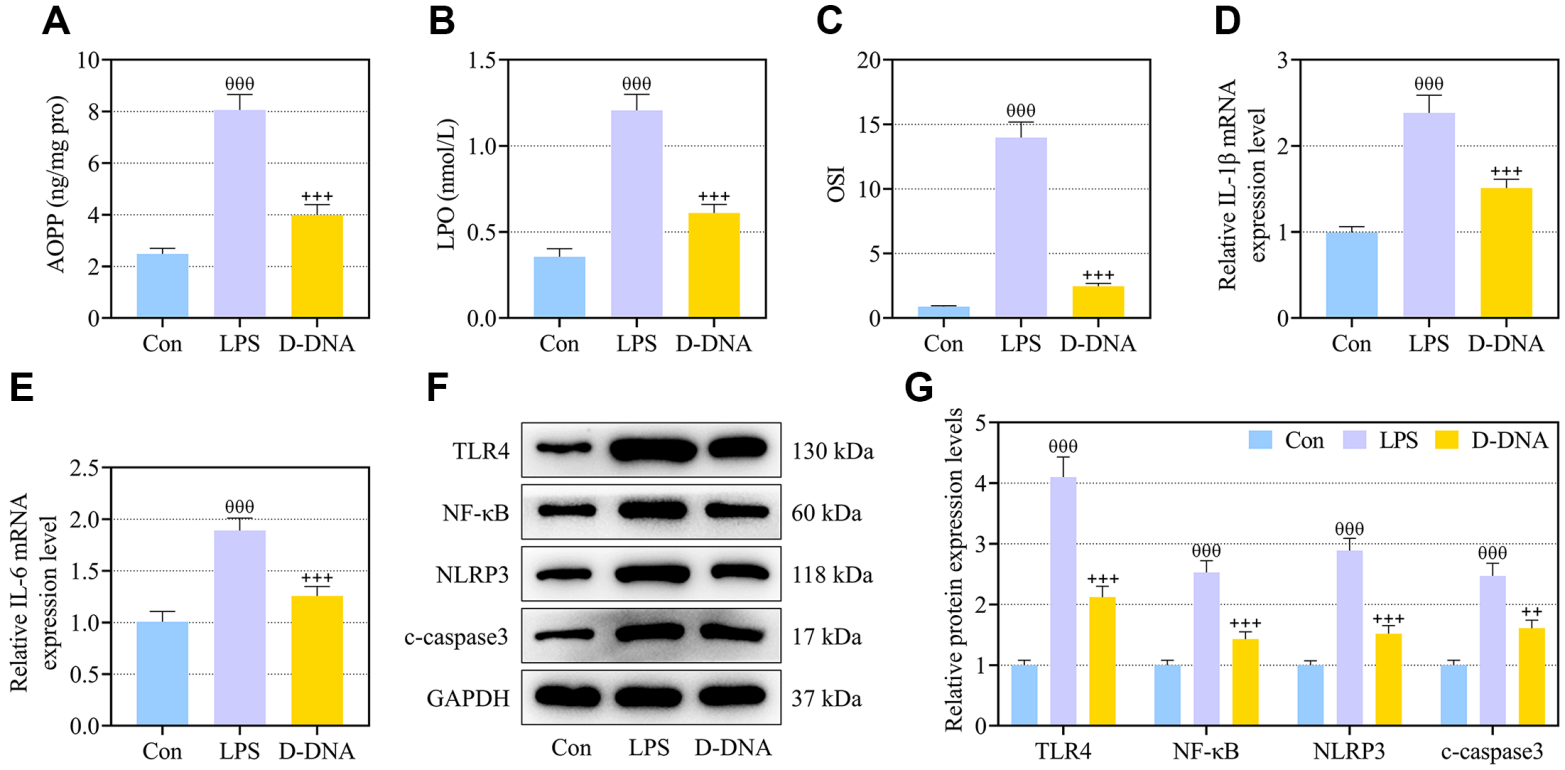
D-DNA pretreatment inhibited lipid peroxidation, inflammation and TLR4/NF-κB signaling in LPS-exposed IEC-6 cells. (**A-G**) IEC-6 cells were exposed to LPS for 1 h before a 1-h administration with 75 μg/ml DNA from DS0384 (D-DNA). (**A-C**) The levels of AOPP and LPO in IEC-6 cells were measured via the colorimetric method and OSI was calculated. (**D, E**) The expressions of IL-1β and IL-6 in IEC-6 cells were analyzed using qRT-PCR, with GAPDH serving as the internal control. (**F, G**) The expressions of TLR4, NF-κB, NLRP3 and c-caspase3 in IEC-6 cells were analyzed using western blot, with GAPDH serving as the reference gene. ^θθθ^ vs. Con, *p* < 0.001; ^++^ vs. LPS, *p* < 0.01, ^+++^ *p* < 0.001. (NEC, necrotizing enterocolitis; LPS, lipopolysaccharide; AOPP, advanced oxidation protein products; LPO, lactoperoxidase; OSI, oxidative stress index; IL-1β/IL-6, interleukin-1β/interleukin-6; TLR4, Toll-like receptor 4; NF-κB, nuclear factor kappa-B; NLRP3, NLR family pyrin domain containing 3; qRT-PCR, quantitative reverse transcription polymerase chain reaction).

IEC-6 cells exposed to LPS were detected by colorimetric assay to show augmented release of AOPP and LPO and the level of OSI, whereas D-DNA pretreatment counteracted the LPS-induced augmentative effect on the release/level of these lipid peroxidation-related markers (Fig. 4A-4C, *p* < 0.001). IL-1β and IL-6 expressions mounted with LPS stimulation in IEC-6 cells, which was partly neutralized by the addition of D-DNA (Fig. 4D and 4E, *p* < 0.001) as presented by qRT-PCR. Western blot detected elevated expressions of TLR4, NF-κB, NLRP3 and c-caspase3 in IEC-6 cells after exposure to LPS (Fig. 4F and 4G, *p* < 0.001). D-DNA pretreatment was discovered to curb the LPS-caused elevation of TLR4, NF-κB, NLRP3 and c-caspase3 expressions in IEC-6 cells (Fig. 4F and 4G, *p* < 0.01).

### FASN or TLR4 Overexpression Abolished D-DNA-Induced Effect on Viability and Apoptosis of LPS-Exposed IEC-6 Cells

**Fig. 5:**
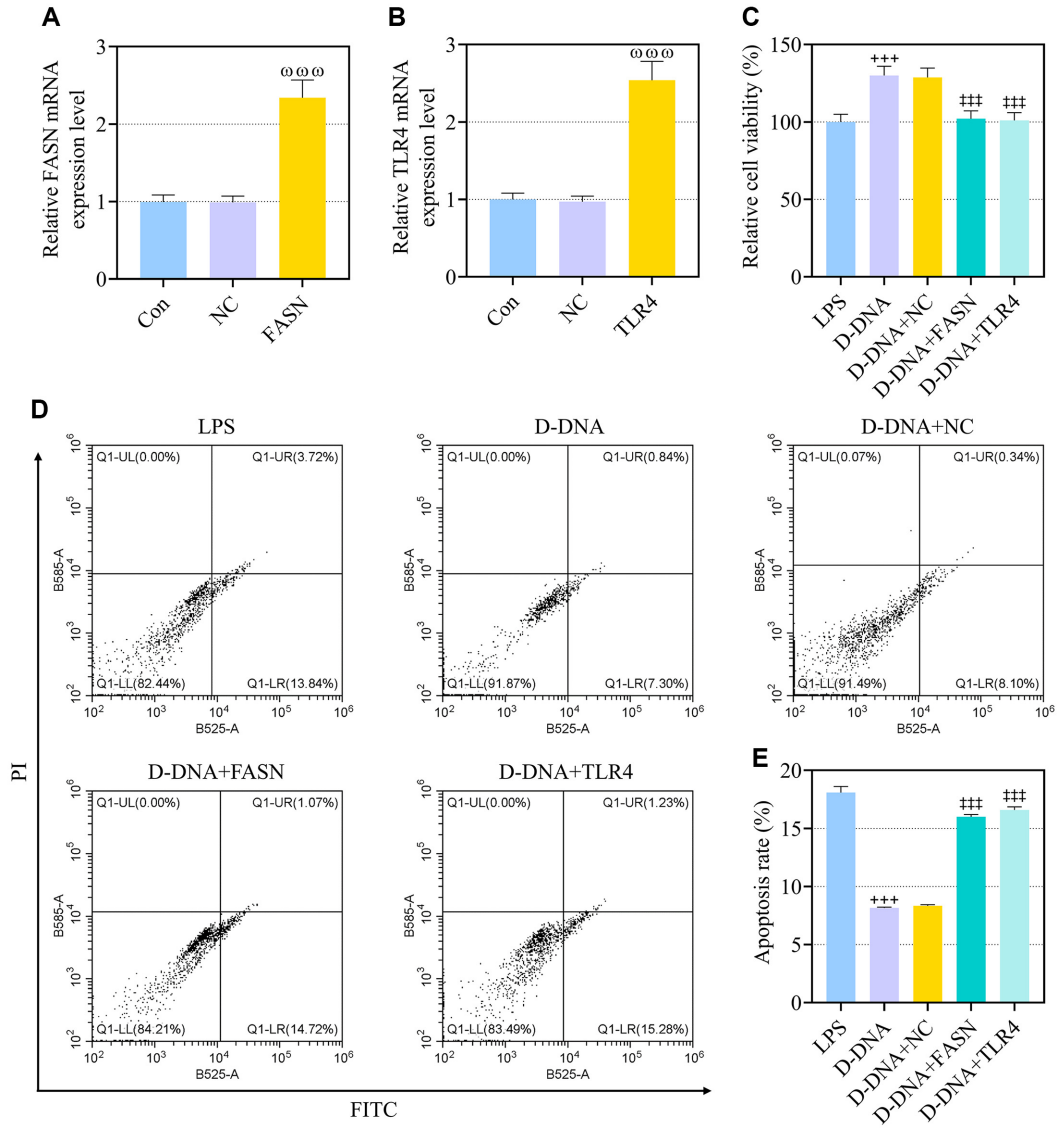
FASN or TLR4 overexpression abolished D-DNA-induced effect on viability and apoptosis of LPS-exposed IEC-6 cells. (**A, B**). The expressions of FASN and TLR4 in IEC-6 cells transfected with FASN and TLR4 overexpression plasmids respectively were analyzed using qRT-PCR, with GAPDH serving as the internal control. (**C-E**) IEC-6 cells received transfection with FASN or TLR4 overexpression plasmids, and were exposed to LPS for 1 h before a 1-h administration with 75 μg/ml DNA from DS0384 (D-DNA). (**C**) The viability of IEC- 6 cells was measured by cell counting kit-8 assay. (**D, E**) The apoptosis of IEC-6 cells was detected through flow cytometry. ^ωωω^ vs. NC, *p* < 0.001; ^+++^ vs. LPS, *p* < 0.001; ^‡‡‡^ vs. D-DNA+NC, *p* < 0.001. (NEC, necrotizing enterocolitis; LPS, lipopolysaccharide; TLR4, Toll-like receptor 4; PI, propidium iodide; FITC, fluorescein isothiocyanate; NC, negative control; qRT-PCR, quantitative reverse transcription polymerase chain reaction).

Transfection with plasmids overexpressing FASN and TLR4 led to over expressions of these two genes (Fig. 5A and 5B, *p* < 0.001). D-DNA-delivered rescue of the viability of LPS-induced IEC-6 cells almost disappeared when FASN or TLR4 overexpression was achieved in LPS-induced IEC-6 cells (Fig. 5C, *p* < 0.001). FASN or TLR4 overexpression was also found to abolish the D-DNA-induced inhibition of LPS-elicited IEC-6 cell apoptosis (Fig. 5D and 5E, *p* < 0.001).

### FASN or TLR4 Overexpression Resisted D-DNA-Delivered Effect on FASN Expression, Lipid Peroxidation, Inflammation and TLR4/NF-κB Signaling in LPS-Exposed IEC-6 Cells

**Fig. 6:**
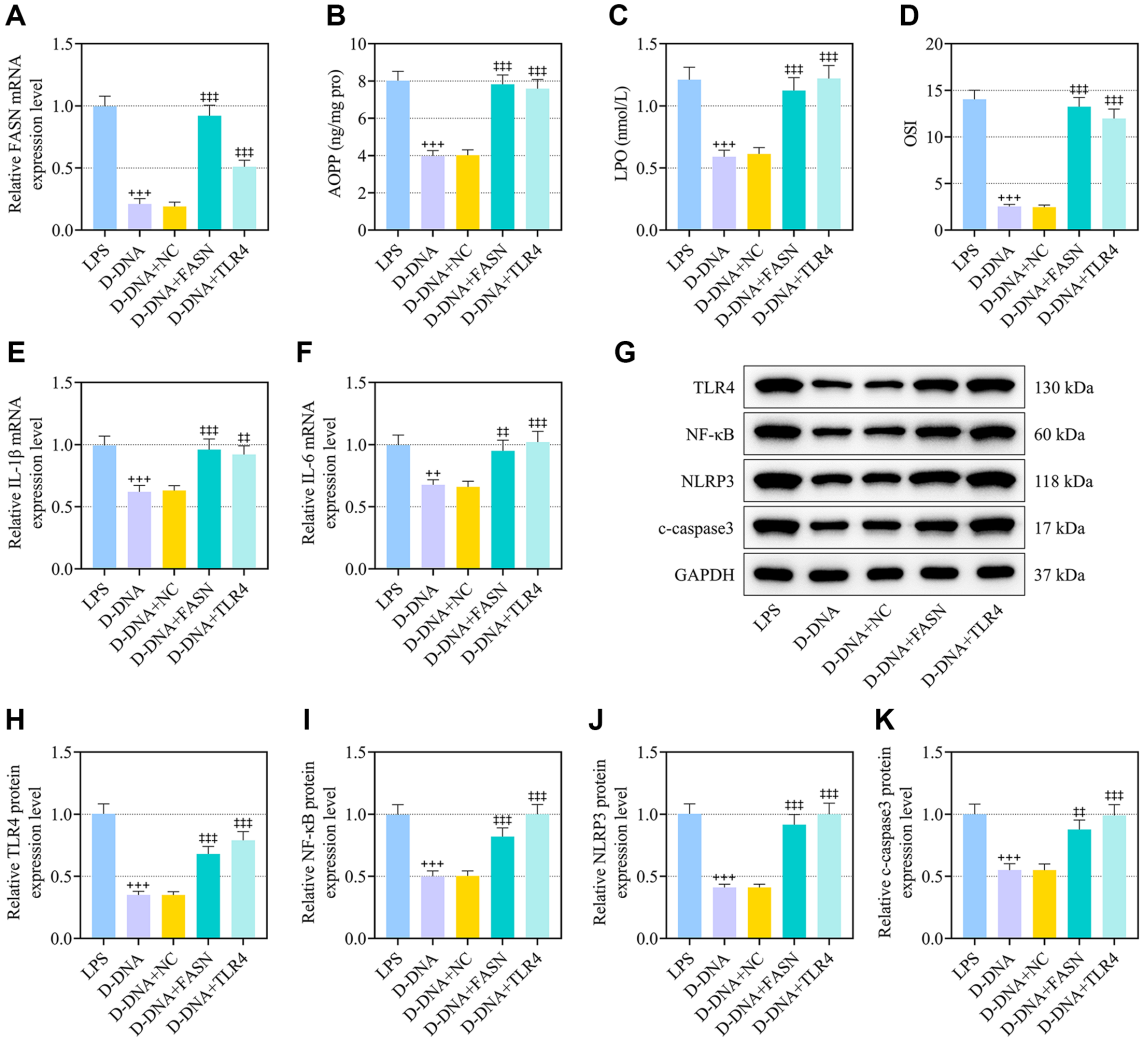
FASN or TLR4 overexpression resisted D-DNA-delivered effect on FASN expression, lipid peroxidation, inflammation and TLR4/NF-κB signaling in LPS-exposed IEC-6 cells. (**A-K**) IEC-6 cells received transfection with FASN or TLR4 overexpression plasmids, and were exposed to LPS for 1 h before a 1-h administration with 75 μg/ml DNA from DS0384 (D-DNA). (**A**) The expression of FASN in IEC-6 cells was analyzed using qRT-PCR, with GAPDH serving as the internal control. (**B-D**) The levels of AOPP and LPO in IEC-6 cells were measured via the colorimetric method and OSI was calculated. (**E, F**) The expressions of IL-1β and IL-6 in IEC-6 cells were analyzed using qRT-PCR, with GAPDH serving as the internal control. (**G-K**) The expressions of TLR4, NF-κB, NLRP3 and c-caspase3 in IEC-6 cells were analyzed using western blot, with GAPDH serving as the reference gene. ^++^ vs. LPS, *p* < 0.01, ^+++^ *p* < 0.001; ^‡‡^ vs. D-DNA+NC, *p* < 0.01, ^‡‡‡^ *p* < 0.001. (NEC, necrotizing enterocolitis; LPS, lipopolysaccharide; AOPP, advanced oxidation protein products; LPO, lactoperoxidase; OSI, oxidative stress index; IL-1β/IL-6, interleukin-1β/interleukin-6; TLR4, Toll-like receptor 4; NF-κB, nuclear factor kappa-B; NLRP3, NLR family pyrin domain containing 3; NC, negative control; qRT-PCR, quantitative reverse transcription polymerase chain reaction).

D-DNA pretreatment repressed FASN expression in LPS-stimulated IEC-6 cells, which was partly lifted by FASN or TLR4 overexpression (Fig. 6A, *p* < 0.001). The secretion of AOPP, LPO and the augment of the OSI level by LPS in IEC-6 cells were suppressed by D-DNA pretreatment, and FASN or TLR4 overexpression abrogated this suppressive effect induced by D-DNA (Fig. 6B-6D, *p* < 0.001). D-DNA-delivered neutralization of IL-1β and IL-6 expressions in LPS-stimulated IEC-6 cells went almost invalid once FASN or TLR4 was overexpressed (Fig. 6E and 6F, *p* < 0.01). FASN or TLR4 overexpression lifted the D-DNA-induced restraint of LPS-posed upregulation of TLR4, NF-κB, NLRP3 and c-caspase3 expressions in IEC-6 cells (Fig. 6G-6K, *p* < 0.01).

## Discussion

NEC is the leading cause of morbidity and mortality in premature neonates, and its development is associated with gut microbiota dysbiosis, which causes intestinal epithelial barrier dysfunction [6]. In the current context where no specific treatment for NEC has been proposed [2], the regulation of gut microbiota to prevent barrier dysfunction may emerge as a promising method treating NEC.

The early signs of NEC are nonspecific and insidious but can rapidly progress to intestinal perforation [22]. In NEC ileal lesions, enterocyte apoptosis is elicited owing to impaired management of oxidative stress in the presence of bacterial TLR4 activation in NEC [4], and it was also detected in our NEC mice, as evidenced by caspase3 positive [23]. DS0384 is isolated from *L. reuteri*, which exerts a protective effect aganist NEC in newborn preterm piglets [12]. Besides, DS0384 has been previously found to be conducive to intestinal regeneration by stimulating intestinal stem cell proliferation in human intestinal organoids and facilitating intestinal maturation in infant mice [13]. Further, *Lactobacillus rhamnosus* HN001 or its DNA can be used as a novel agent to prevent NEC in humans, acting in part through the microbial DNA receptor TLR9 [18]. Our study revealed that DS0384/D-DNA directly diminished ileal lesions accompanied by reduced cell apoptosis in NEC mice as well as inhibiting epithelial cell apoptosis in *in vitro* NEC, which suggests that transplantation with DS0384 into premature mice antagonizes NEC development and progression. However, further research needs to be done to answer the exact mechanism of how DS0384 probiotics actually function.

Interestingly, NCG, a metabolite of DS0384, is held accountable for the beneficial effect of DS0384 on intestinal maturation [13]. NCG is a analogue of N-acetylglutamate, which promotes the synthesis of endogenous Arg [24, 25], and it has been used as a feed additive with safety, metabolic stability and higher absorption rate [26]. Lipid peroxidation of membrane is induced by preterm insult-triggered oxidative stress and inflammation [4, 27], resulting in enhanced inflammatory responses, damaged collagen and mucosal basement membrane, cell degeneration and necrosis in NEC [28]. Of note, supplementation with NCG makes for the reduction of hepatocyte and adipocyte apoptosis and the amelioration of inflammation, lipid metabolism in Japanese seabass as well as elevates the antioxidant status [16, 24, 29, 30]. Our result showed that the content of NCG was found to be low in the ileum of our NEC mice, while being restored after treatment with DS0384. Meanwhile, the robust pro-inflammatory mucosal immune response in NEC, as reflected by lifted *in-vivo* and *in-vitro* levels of the inflammatory cytokines, IL-1β and IL-6, dampened with DS0384/D-DNA treatment. These above findings hint that NCG may also plays a crtical role in the DS0384-induced alleviation of NEC by inhibiting lipid peroxidation and inflammation.

Moreover, FASN, the central regulator of lipid metabolism, is a large multi-enzyme complex those functions to produce saturated fatty acid, palmitate, with the aid of NADPH [31]. Our study detected that the addtion of DS0384 led to repression of the NEC-caused upregulation of FASN and lipid peroxidation-related markers, AOPP, LPO and OSI. This result combined with the previous data confirming that NCG decreases FASN expression [16] suggests that FASN represented the main target for the effect of NCG secreted by DS0384 in NEC. Besides, TLR4-mediated pro-inflammatory responses featuring upregulation of TLR4 in the intestinal mucosa is a typical hallmark in NEC pathogenesis [32]. Decreased TLR4 expression correlates with reduced NEC severity [33], which displays alleviated intestinal epithelial barrier injury with an increased expression of ZO-1 [34, 35], a tight junction protein required for junction assembly [36]. NCG downregulates TLR4 to suppress inflamamtion, thereby enhancing duodenal barrier function in suckling lambs suffering from growth-retardation [37]. Our study showed that DS0384 resisted TLR4 or FASN upregulation and ZO-1 downregulation induced by NEC in the mice’s ileum. Taken together, DS0384 was thought to downregulate TLR4 or FASN to rescue the function of the intestinal epithelial barrier in NEC. However, subsequent experiments did not emphasize the role of DS0384 in regulating ZO-1 expression, which is a limitation of this study, more experiment are need to verify.

The interaction of TLR4 with LPS initiates myeloid differentiation factor MyD88-mediated signaling cascades to activate the downstream NF-κB, which further induces the transcriptions of inflammatory cytokines [38]. Furthermore, NLRP3, an intracellular signalling molecule, is activated after sensing many pathogen/environmental/host-derived factors [39] including inflammation-related activation of TLR4-NF-κB signaling [40]. Previous study showed that FASN activates the NF-κB/STAT3 signaling pathway by phosphorylation of upstream IKKα and i-κBα. In our study, overexpression TLR4 or FASN reversed the inhibition effect of D-DNA on TLR4, NF-kB, NLRP3 and c-caspase3 expression in NEC IEC-6 cells, indicating that NF-κB-mediated NLRP3 activation is involved in the mitigation of NEC intestinal epithelial barrier damage by D-DNA. However, further validation with NF-kB pathway inhibitors and pathway inducers is needed in the future.

TLR4 inhibition results in the mitigation of lipid peroxidation [15]. Products of lipid peroxidation exacerbate colonic inflammation [41]. FASN-dependent MYD88 palmitoylation is required in toll-like receptor-mediated inflammation [42]. Similarily, in our study, FASN overexpression in D-DNA-treated NEC IEC-6 cells restored TLR4 expression as well as upergulating the level of inflammatory cytokines, promoting NF-κB-mediated NLRP3 activation and enhancing apoptosis. Dampening TLR4 activation reduces lipid peroxidation in NEC [5, 43]. We discovered that when TLR4 was overexpressed, NEC IEC-6 cells treated with D-DNA showed higher levels of FASN and OSI and secreted more AOPP and LPO, which indicates that TLR4 activation contibutes to lipid peroxidation in the NEC intestinal epithelial barrier. However, the effective dosage and concentration of D-DNA not detected is the limitation of this study. Moreover, arginine supplementation is beneficial in reducing fat deposition, NCG has been proven to increase endogenous arginine synthesis and arginine level in circulating blood [16, 29]. Arginine inhibited the TLR4 downstream pathway by binding to TLR4 and consequently activated Janus kinase (JAK) 2/signal transducer [44]. Inhibition of JAK/STAT signaling pathway alleviates ulcerative colitis [45]. This remind us that NCG affects the TLR4 pathways by regulating arginine and JAK, which need more experiment to verify.

In conclusion, the present study demonstrated that supplement with DS0384 serves as gut microbiota intervention that subdues lipid peroxidation and inflammation to alleviate NEC by regulating FASN and TLR4 expression.

## Supplemental Materials

Supplementary data for this paper are available on-line only at http://jmb.or.kr.
