# Supplementary material for: DS0384 Alleviates Necrotizing Enterocolitis: Secretes N-carbamyl glutamic Acid and Participates in Lipid Metabolism and Lipid Peroxidation Processes
Source: J Microbiol Biotechnol. 2025 Feb 13;35:e2410040. doi: 10.4014/jmb.2410.10040 (PMC11879329; doi:10.4014/jmb.2410.10040)
Supplement: Supplementary file 1 [file jmb-35-e2410040-supple.pdf]

J. Microbiol. Biotechnol. 2025. 35: e2410040
https://doi.org/10.4014/jmb.2410.10040

Table 1: Primers used in quantitative reverse transcription polymerase chain reaction for related genes.

Genes	Species	Forward (5’- -3’)	Reverse (5’- -3’)
FASN	mouse	GGAGGTGGTGATAGCCGGTAT	TGGGTAATCCATAGAGCCCAG
	rat	AAGCCCTTTCCAGCCCATAC	AGCCAGGTCCCTCTAGTGAG
ACC1	mouse	GATGAACCATCTCCGTTGGC	GACCCAATTATGAATCGGGAGTG
	rat	GACAAAGGTCTGGCCACGTA	ACAAACCCAACTGGCCTCAA
ATGL	mouse	GGATGGCGGCATTTCAGACA	CAAAGGGTTGGGTTGGTTCAG
	rat	CTCCTCTCCCTGGCTACTGT	ATACTTAGGACGCCCTGGGT
HSL	mouse	GATTTACGCACGATGACACAGT	ACCTGCAAAGACATTAGACAGC
	rat	GGGGGTGGGAATTGGCATTA	CCCTCTTCCCCAGAAAGCTG
IL-1β	mouse	GCAACTGTTCCTGAACTCAACT	ATCTTTTGGGGTCCGTCAACT-3’
	rat	GTCCCCAATCCTGTCCCTTG	TCCGTACAAGGAACCGTGTG
IL-6	mouse	TAGTCCTTCCTACCCCAATTTCC’	TTGGTCCTTAGCCACTCCTTC
	rat	ATCTGCCCTTCAGGAACAGC	CTCAATAGCTCCGCCAGAGG
GAPDH	mouse	AGGTCGGTGTGAACGGATTTG	TGTAGACCATGTAGTTGAGGTCA
	rat	TGGATAGGGTGGCCGAAGTA	TACAAGGGGAGCAACAGCTG
